# Supplementary material for: Adherence to Menzerath's Law is the exception (not the rule) in three duetting primate species
Source: R Soc Open Sci. 2020 Nov 18;7(11):201557. doi: 10.1098/rsos.201557 (PMC7735330; doi:10.1098/rsos.201557)
Supplement: Data collection and acoustic analysis protocols for all datasets included in the present analysis. [file rsos201557supp1.docx]

**Clink and Lau Electronic Supplementary Material 1. Data collection and acoustic analysis protocols for all datasets included in the present analysis.**

**Tarsiers** [1]

Data collection

Data were collected July and August 2018 in Tangkoko National Park, Sulawesi, Indonesia. We collected data using a combination of focal recordings and autonomous recorders. For focal recordings, we used a RØDE NT-USB Condenser Microphone (Røde Microphones, Sydney, Australia) connected to a 32 GB Apple iPad Air (Apple Inc., Cupertino, CA) and the Voice Record Pro application at a sampling rate of 44.1 kHz and 16 bits. We used 2 different autonomous recording devices, an ARBIMON portable recorder which recorded at 44.1 kHz and 16 bits, or a SWIFT recorder which recorded at 48 kHz and 16 bits.

Acoustic analysis

We downsampled the data with a 48 kHz sampling to a sampling rate of 44.1 kHz using Audacity(R) 2.3.0 software (2018). We created spectrograms using Raven Pro 1.5 Sound Analysis Software. Spectrograms were created with a 1600- point (33.3 ms) Hann window (3 dB bandwidth . 43.1 Hz), with 50% overlap, and a 2048-point discrete Fourier transform, yielding time and frequency measurement precision of 16.7 ms and 24.1 Hz.

**Titi monkeys** [2,3]

Data collection

Titi monkey duets were recorded opportunistically at the California National Primate Research

Center (CNPRC), Davis, CA, USA each morning between 06:00 and 07:30 from March 2017 to March 2019. We used a Marantz PMD 660 flash recorder and a Marantz Professional Audio Scope SG-5B directional condenser microphone. We recorded at a sampling rate of 44.1 kHz and 16-bit resolution.

Acoustic analysis

We created spectrograms using Raven Pro 1.5 Sound Analysis Software. We generated spectrograms with a 512-point (11.6 ms) Hann window (3 dB bandwidth = 124 Hz), with 75% overlap, and a 1024-point DFT, yielding time and frequency measurement precision of 2.9 ms and 43.1 Hz.

**Gibbon duets (female introduction and trills; male codas** [4,5]**)**

Data collection

We collected recordings from gibbons at seven sites across Sabah, Malaysia during multiple field seasons from January 2013 to September 2016. We recorded

vocalizations at a sampling rate of 44.1 kHz and 16-bit size using a Marantz PMD 660 flash recorder (Marantz, Kawasaki, Kanagawa Prefecture, Japan) equipped with a

Røde NTG-2 directional condenser microphone (Røde Microphones, Sydney, Australia).

Acoustic analysis

We created spectrograms using the Raven Pro 1.5 sound analysis with a 512-point (11.6 ms) Hann window (3 dB bandwidth ± 124Hz), with 75% overlap, and a 1024-point discrete Fourier transform, yielding time and frequency measurement precision of 2.9ms and 43.1 Hz. For the female calls we used the band-limited energy detector in Raven Pro to identify individual notes from each great call.

**Gibbon male solos** [6]

Data collection

We collected data using Swift autonomous recording units in Danum Valley Conservation Area (11 recording units; March–July 2018) and in Maliau Basin Conservation Area (four recording units; August 2019), Sabah, Malaysia. The units in Danum Valley Conservation Area recorded at a sampling rate of 16 kHz and the units in Maliau Basin recorded at a sampling rate of 48 kHz. For both recording locations, we recorded at a sample size of 16 bits and at a gain of 40 dB.

Acoustic analysis

We created spectrograms in Raven Pro 1.6.We downsampled the 48 kHz recordings to 16 kHz. We then made spectrograms with a 1024-point (64.0 ms) Hann window (3 dB bandwidth = 22.5 Hz), with 50% overlap, and a 1024-point discrete Fourier transform, yielding time and frequency measurement precision of 32 ms and 15.6 Hz.

**Works Cited**

1. Clink DJ, Tasirin JS, Klinck H. 2019 Vocal individuality and rhythm in male and female duet contributions of a nonhuman primate. *Curr. Zool.* **0**, 1–14.

2. Clink DJ, Lau AR, Bales KL. 2019 Age-related changes and vocal convergence in titi monkey duet pulses. *Behaviour* **156**, 1471–1494. (doi:10.1163/1568539X-00003575)

3. Lau AR, Clink DJ, Bales KL. 2020 Individuality in the vocalizations of infant and adult coppery titi monkeys (Plecturocebus cupreus). *Am. J. Primatol.* (doi:10.1002/ajp.23134)

4. Lau A, Clink DJ, Crofoot MC, Marshall AJ. 2018 Evidence for High Variability in Temporal Features of the Male Coda in Müller’s Bornean Gibbons (*Hylobates muelleri*). *Int. J. Primatol.* **39**, 670–684.

5. Clink DJ, Grote MN, Crofoot MC, Marshall AJ. 2018 Understanding sources of variance and correlation among features of Bornean gibbon (*Hylobates muelleri*) female calls. *J. Acoust. Soc. Am.*

6. Clink DJ, Hamid Ahmad A, Klinck H. 2020 Brevity is not a universal in animal communication: evidence for compression depends on the unit of analysis in small ape vocalizations. *R. Soc. Open Sci.* **7**.
